# Stigmatizing attitudes of Swiss youth towards peers with mental disorders

**DOI:** 10.1371/journal.pone.0235034

**Published:** 2020-07-24

**Authors:** Michelle Dey, Raquel Paz Castro, Anthony Francis Jorm, Laurent Marti, Michael Patrick Schaub, Andrew Mackinnon

**Affiliations:** 1 Swiss Research Institute for Public Health and Addiction, University of Zurich, Zurich, Switzerland; 2 Centre for Mental Health, Melbourne School of Population and Global Health, University of Melbourne, Melbourne, Victoria, Australia; 3 Yunus Centre for Social Business and Health, Glasgow Caledonian University, Glasgow, Scotland, United Kingdom; 4 Black Dog Institute, University of New South Wales, Sydney, Australia; University of the Witwatersrand, SOUTH AFRICA

## Abstract

**Background:**

Previous research on public stigma towards people with mental disorders has mostly targeted adult samples and focused on depression, schizophrenia or mental disorders in general. Hence, the present study aimed to investigate predictors of stigmatizing attitudes towards different mental disorders (including less researched ones) in a representative sample of adolescents and young adults.

**Methods:**

Data from the Swiss Youth Mental Health Literacy and Stigma Survey were used (analytical sample: n = 4,932). Each participant was randomly presented with one of five vignettes (depression; alcohol abuse; depression and alcohol abuse combined; schizophrenia; social anxiety). The structure of stigmatizing attitudes was assessed using confirmatory factor analysis. Regression models, implemented within a structural equation framework, were used to study predictors for the identified latent variables.

**Results:**

A three-factor model for stigmatizing attitudes–consisting of ‘dangerous/unpredictable’, ‘weak-no-sick’, and ‘social distance’ factors–best fitted the data. Female gender was associated with less stigmatizing attitudes. Associations in opposite directions with different latent factors were found for educational and migration background. Exposure to mental disorders (being personally affected, personally having received professional help or knowing someone close who has received treatment for a mental disorder) was either not or was negatively associated with stigmatizing attitudes. In contrast, current mental health symptoms (heightened levels of psychological distress, problematic alcohol use) were generally not or were positively associated with stigmatizing attitudes. Even though the included predictors had some predictive value, the variance explained by the models was rather small (the adjusted R^2^ varied between 0.03 and 0.26).

**Conclusions:**

The current study indicates that contact with someone who has received treatment for a mental disorder might be an important component of programs aiming to decrease stigmatizing attitudes towards people with mental disorders, since this exposure variable predicted lower levels of stigmatizing attitudes. Furthermore, the findings suggest that target-group interventions for specific subgroups need to be considered, as the process leading to stigmatizing attitudes towards people with mental disorders appears to differ between specific sociodemographic subgroups.

## Introduction

Many people with mental disorders (including substance-related disorders) experience a ‘double burden’, as they are not only affected by their condition, but also face stigmatization [1, 2]. The stigma of mental illness results in decreased life opportunities and a loss of independent functioning [1, 3]. Furthermore, stigma constitutes a major barrier to seeking help for mental disorders [4, 5]. This is problematic insofar as forgone or delayed care further exacerbates the affected person’s condition and living situation [6].

The current article focuses on a subtype of public stigma called *personal stigma*, which describes a person’s individual attitudes towards people with mental disorders [7]. However, since not all researchers distinguish between the two components of public stigma, i.e. personal and perceived stigma (defined as a person’s perception of what most other people believe about a particular group; [7])–the superordinate term ‘public stigma’ will generally be used subsequently. Public stigma (defined as the general public’s attitudes towards people with mental disorders) must be differentiated from self-stigma, which is characterized by the process of turning prejudice against oneself among individuals with mental disorders [1].

In order to plan tailored programs aiming at reducing public stigma in the general population and thereby also improve help seeking by affected individuals, it is important to identify factors associated with heightened levels of stigmatizing attitudes. In this regard, various sociodemographic variables have been proposed as predicting or being associated with having stigmatizing attitudes. These include older age [3, 8, 9] and lower educational attainment [9, 10]. Furthermore, exposure to mental disorders (also labelled as familiarity or contact), which has mostly been operationalized as having had personal experiences with mental health problems or knowing someone with such a condition, has been examined repeatedly [9–11]. Two reviews of studies that mainly sampled adults suggest that such an exposure is associated with a higher acceptance of people with mental disorders [9] and with less desire for social distance from the person with the mental illness [10]. However, a more recent review suggested a qualified U-shaped association between familiarity, with a negative relationship between familiarity and stigma at lower levels of familiarity, and a positive relationship at higher levels [11]. The latter indicates that exposure to mental disorders may–in some groups (e.g. nuclear family, service providers)–lead to greater public stigma. Lastly, a review that specifically focused on youth concluded that the association between exposure and stigmatizing attitudes is not unequivocal [8]. The inconsistencies between the above review articles might–besides different sample characteristics (e.g., in terms of the targeted age group)–stem from the particular mental disorders that were considered in the included studies, since different mental health conditions are stigmatized to different extents [9, 10, 12, 13]. Furthermore, some studies used a stigma measure that included items about personal *and* perceived stigma, even though some predictors seem to be associated with these types of stigma in opposite directions. Exposure, for instance, has been shown to be associated with higher perceived, but lower personal stigma [14].

Different gaps regarding research on public stigma towards people with mental disorders must be considered. Most importantly, research in this field is limited in mainly focusing on adult samples and predominantly focusing on schizophrenia, depression or mental disorders non specifically [9, 15–17]. Furthermore, current mental health symptoms have only rarely been included as predictors of stigmatizing attitudes. In order to address these research gaps, the current article aimed to study predictors of stigmatizing attitudes towards a broad range of mental disorders, including the less-researched conditions of social anxiety and alcohol abuse, in a representative sample of adolescents and young adults. Furthermore, this study considered measures pertaining to current mental health symptoms–i.e. psychological distress and problematic alcohol use–alongside demographic and exposure variables to predict stigmatizing attitudes.

## Materials and methods

### Procedures and sample

Data from the Swiss Youth Mental Health Literacy and Stigma Survey (SYMHLSS) were used for the current analysis. Details of the procedure of the survey have been described elsewhere [18]. In short, the SYMHLSS was adapted from the Australian National Survey of Youth Mental Health Literacy and Stigma [19, 20]. In Switzerland, the study was carried out as a school-based survey between October 2017 and June 2018. The target population consisted of students at the upper secondary educational level in German-speaking parts of Switzerland (corresponding to ISCED3). A two-stage stratified sample design was used to ascertain this population. The first-stage sampling units were individual schools, the second-stage sampling units were classes within these schools (generally, three classes were sampled from each school).

In Switzerland, mandatory schooling is typically concluded around the age of 15 years. Subsequently, two educational streams are available: The *vocational education track (VET)* takes up to four years to conclude (depending on the specific vocation) and combines an apprenticeship (i.e., workplace-based training at the site of an employer) and school-based learning. During or after concluding an apprenticeship, a vocational baccalaureate program can be attended with the aim of deepening the basic training provided by vocational schools. Participants who were pursuing such a vocational baccalaureate were also included in the current study. They were typically older relative to other participants. The second academic stream is called *general education track (GE)* and includes secondary schooling. This track is typically roughly completed around the age of 19. VET- and GE-schools were represented proportionally in the current study.

All students in a class were asked to participate in the survey. Research staff introduced the survey to the students at the beginning of a school lesson and those pupils who were willing to participate (i.e. who provided written informed consent) filled out the online questionnaire during the remaining time. Altogether, 4,983 students participated in the survey, corresponding to a response rate of 99.4%. The study protocol was approved by the Ethics Committee of the University of Zurich (approval number: 17.4.9). This committee granted approval on the basis that there was no need for guardians’ consent for participation.

### Questionnaire

The core elements of the questionnaire comprised vignettes describing a young person with a mental disorder. Five different vignettes were used in the SYMHLSS, namely depression (DEP), alcohol abuse (ALC), alcohol abuse and depression combined (ALC & DEP), schizophrenia (SCH) and social anxiety (SOC). One of these five vignettes was presented randomly to each participant (the randomisation of the vignettes was implemented in the programmed online version of the questionnaire). The character in the vignette was called Lukas (for male participants) or Anna (for female participants) and was described as being about the same age as the participant. This gender- and age-matching of the character in the vignette and the participant’s characteristics was implemented with the aim of ensuring that the participant could optimally relate to the adolescent described in the vignette. Most subsequent questions–including those about stigmatizing attitudes–referred to these vignettes. The vignettes and subsequent questions are provided verbatim in the study protocol [18].

#### Questions regarding stigma

Two blocks of stigma questions were asked. In the first block, participants had to indicate how strongly they personally agreed/disagreed with eight statements (e.g., ‘Anna’s / Lukas’s problem is a sign of personal weakness’ or ‘Anna / Lukas is dangerous’). The answer format for these stigma items was re-coded for the analyses and ranged from 0 ‘strongly disagree’ to 4 ‘strongly agree’. The second block asked participants how happy they would be to spend time with Anna / Lukas in five situations with different levels of personal closeness (e.g., working on a project or developing a close friendship with Anna / Lukas). The answer format of these questions ranged from 0 ‘yes, definitely’ to 3 ‘definitely not’. In both blocks, higher scores indicated higher levels of stigmatizing attitudes.

#### Predictors for stigmatizing attitudes

The *demographic variables*, age, gender, academic track (VET vs. GE) and migration background (‘Swiss origin’ vs. ‘two-sided migration background’, i.e. both female and male caregivers were born in a country other than Switzerland) were used as predictors.

In addition, variables reflecting *current mental health symptoms* were considered. Current mental health status was assessed with the *Kessler Psychological Distress Scale (K6)*, which measures psychological distress [21, 22]. Referring to the past 30 days, participants were asked about the frequency of having felt i) nervous, ii) hopeless, iii) restless or fidgety, iv) so depressed that nothing could cheer them up, v) that everything was an effort, and vi) worthless. A five-point Likert scale was used, ranging from 0 ‘none of the time’ to 4 ‘all of the time’. For the analyses, a sum score that could range from 0 to 24 was used. The *AUDIT-C* [23, 24] was used to assess problematic alcohol use. This instrument consists of three questions asking about the frequency and typical quantity as well as risky single-occasion drinking: 1) ‘How often did you have a drink containing alcohol in the past year?’ (answer format: 5-point Likert scale ranging from 0 ‘never’ to 4 ‘4 or more times a week’), 2) ‘How many drinks did you have on a typical day when you were drinking in the past year?’ (answer format: 5-point Likert scale ranging from 0 ‘1 or 2 drinks’ to 4 ‘10 or more drinks’), and 3) ‘How often did you have 6 or more drinks on one occasion in the past year?’ (answer format: 5-point Likert scale ranging from 0 ‘never’ to 4 ‘daily or almost daily’). Scores of four or less were coded as ‘no problematic alcohol use’, five or above as ‘problematic alcohol use’ [24].

Lastly, a number of *exposure/personal experience variables* were included in models. The following questions that were adapted from the Australian National Survey of Youth Mental Health Literacy and Stigma were used: 1) ‘Have you ever had a problem similar to Anna’s / Lukas’s’ (answer format: yes; no; do not want to answer); 2) ‘Have you received any professional help (e.g., from a psychologist, physician) in order to treat this problem?’ (question was only asked when the participant indicated previously that she / he has had a similar problem; answer format: yes; no; do not want to answer); 3) ‘Has anyone in your family or close circle of friends ever had a problem similar to Anna’s / Lukas’s?’ (answer format: yes; no; do not know; do not want to answer) 4) ‘Has this person / any of these people received professional help (e.g., from a psychologist, physician) to treat this problem?’ (question was only asked when the participant indicated previously that a family member / a close friend has had a similar problem; answer format: yes; no; do not know). For all exposure variables, the answer formats ‘no’, ‘do not know’, and ‘do not want to answer’ were collapsed and used as reference category.

### Sample weights

Following standard procedures (see, for example, [25, 26]), the inverse of selection probabilities, in combination with non-response adjustments at both sampling stages, were used to calculate sampling weights. Survey weights ensure that each sampled student represents the appropriate number of students in the population and, hence, allow for the calculation of accurate population estimates and standard errors [26]. These weights were used in the analyses described below.

### Statistical analyses

In a first step, confirmatory factor analysis (CFA) models for the complete data set were conducted to compare three models representing stigma: (1) a model with a single latent variable (i.e. all stigma questions were assumed to load on the same factor), (2) a model with two latent variables (items from the first block loading on a first latent variable ‘prejudice’ and items from the second block on the latent variable ‘social distance’), and (3) a model with three latent variables which differentiated the items in the first block into ‘weak-not-sick’, and ‘dangerous/unpredictable’ latent variables, as derived from previous research [27–29]. In all CFA models, responses to indicators were treated as ordered categories, each with an underlying continuous distribution.

In a second step, regression models, implemented by extending the structural equation measurement models, were constructed for each vignette by adding predictors of the latent variables defined by the CFA. The latent variables that were identified in the CFA were used as outcome variables. Two classes of predictors, which we refer to as *‘initial’* and *‘emerging’*, were defined. Initial predictors were participant attributes present at birth or which are immutable (age, gender, migrant background). These are inherently exogeneous in models. Emerging predictors included other attributes of participants (academic track, indicators of current mental health symptoms and exposure variables) that might be, in part, determined or influenced by the exogeneous predictors and which might or might not add predictive power to models that already included the initial predictors. At first, models that included only the initial predictors were built. Subsequently, the emerging predictors were added to these models. This procedure allowed examining the incremental value of the emerging variables in the prediction of stigmatizing attitudes. The latter models were compared to the initial predictor only models for each vignette using a chi-square test for difference testing. Predictors were manifest. Hence, results are interpreted as being comparable to regression analysis. Data were appropriately weighted for all CFA and prediction models. All models were estimated using Mplus 7.4 [30] using the WLSMV estimator which is appropriate for ordered categorical outcomes and which accommodates participants with missing outcome data. The following goodness-of-fit indices were used to evaluate the models: (1) Chi-square goodness of fit test [31], (2) The DIFTEST chi-square for difference testing (3), Comparative Fit Index (CFI, [32]), (4) Tucker-Lewis Index (TLI, [33]), and (5) the root mean square error of approximation (RMSEA, [34]). CFI and TLI values greater than 0.95 are indicative for a well-fitting model. In the case of the RMSEA, values of ≤0.05 indicate a good fit. For the RMSEA, Mplus additionally tests the hypothesis that RMSEA <0.05 in the population [30]. Values >0.50 indicate a good fit and are also reported [34].

## Results

### Socio-demographic variables

Out of the total sample, 4,932 (99.0%) answered at least one of the stigma questions. This group constituted the analytic sample for the subsequent CFA models. Socio-demographic characteristics and exposure variables for the analytic sample are displayed in Table 1.

**Table 1:** Socio-demographic and exposure characteristics of the analytic sample (N = 4,932).

Variable		
Age, *M (SD)*		17.82 (1.92)
Age categories	17 years or younger	2,514 (51.0%)
	18–20 years	2,031 (41.2%)
	21 years or older	387 (7.8%)
Gender	Female	2,654 (53.8%)
	Male	2,278 (46.2%)
Education track	Vocational	3,583 (72.6%)
	General	1,349 (27.4%)
Migration background	Swiss	2,772 (56.2%)
	One-sided	848 (17.2%)
	Two-sided	1,027 (20.8%)
	Missing	285 (5.8%)
Psychological distress, *M (SD)* ^b^		8.08 (4.76)
Alcohol use ^a^	Non-problematic drinking	3,391 (68.8%)
	Problematic drinking	1,541 (31.2%)
Experienced similar problem	No	3,490 (70.8%)
	Yes	1,199 (24.3%)
	Missing	24 (4.9%)
Received professional treatment	No	805 (16.3%)
	Yes	371 (7.5%)
	NA (no MH problem)	3,756 (76.1%)
Close person experienced similar problem	No	2,185 (44.3%)
	Yes	1,911 (38.7%)
	Missing	836 (13.0%)
Close person received professional treatment	No	730 (14.8%)
	Yes	1,059 (21.5%)
	NA (no MH problem)	3,143 (63.7%)

^a^ Alcohol use was measured by AUDIT-C. Cut-offs for problematic alcohol use were ≥ 5.

^b^ Psychological distress was measured by K6.

### Confirmatory factor analysis

**Fig 1:**
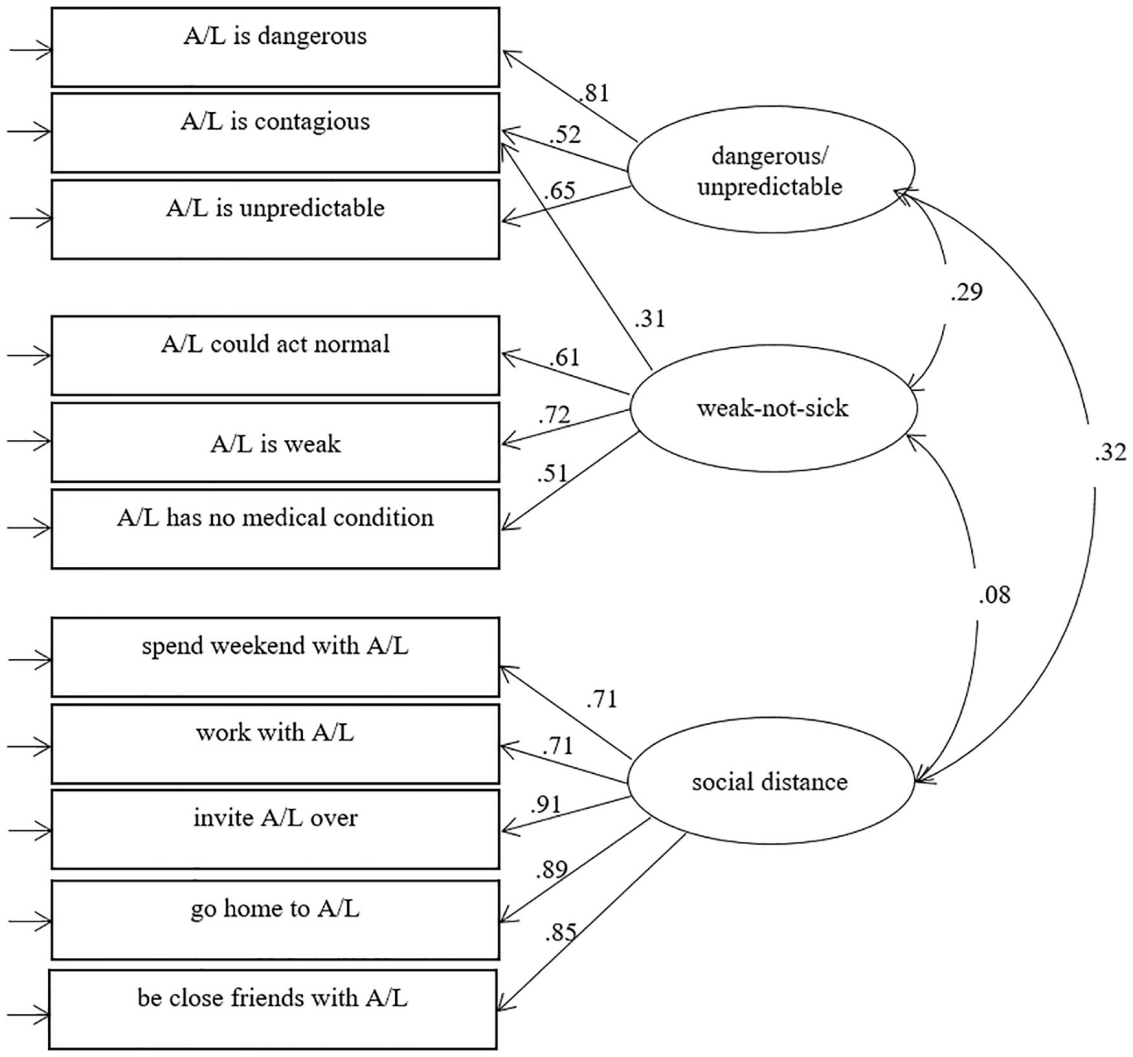
Confirmatory factor model for the 11 stigma items. Standardised coefficients are shown for the model fitted to the total sample (across all five vignettes). All coefficients are statistically significant (p<0.001). A/L = Anna or Lukas.

The models with one factor and two factors indicated an unacceptable fit [1: χ^*2*^ = 6344.87, *df* = 44, *p* < .001; CFI = 0.84; TLI = 0.80; RMSEA = 0.17 (90% CI 0.17–0.17), *p* < .000; 2: χ^*2*^ = 1833.74, *df* = 43, *p* < .000; CFI = 0.95; TLI = 0.94; RMSEA = 0.09 (90% CI 0.09–0.10), *p* < .000]. The model with three factors indicated a better fit in three of four indices compared to the previous models [χ^*2*^ = 658.43, *df* = 41, *p* < .001; CFI = 0.98; TLI = 0.98; RMSEA = 0.06 (90% CI 0.05–0.06), *p* = .010]. To improve fit, the item ‘It is best to avoid (Anna/Lukas) so that you don’t develop this problem yourself’ was permitted to load on two of the two latent variables, namely ‘weak-not-sick’ and ‘dangerous/unpredictable’ (see Fig 1) [χ^*2*^ = 404.42, *df* = 40, *p* < .001; CFI = 0.99; TLI = 0.99; RMSEA = 0.04 (90% CI 0.04–0.05), *p* = .999]. This last three-factor model (Fig 1) formed the basis of the following prediction models. The correlations between the latent variables ranged from negligible (correlation between ‘weak-not-sick’ and ‘social distance’) to small-to-moderate (correlations between ‘dangerous/unpredictable’ and the other latent factors) [35].

### Prediction models

**Fig 2:**
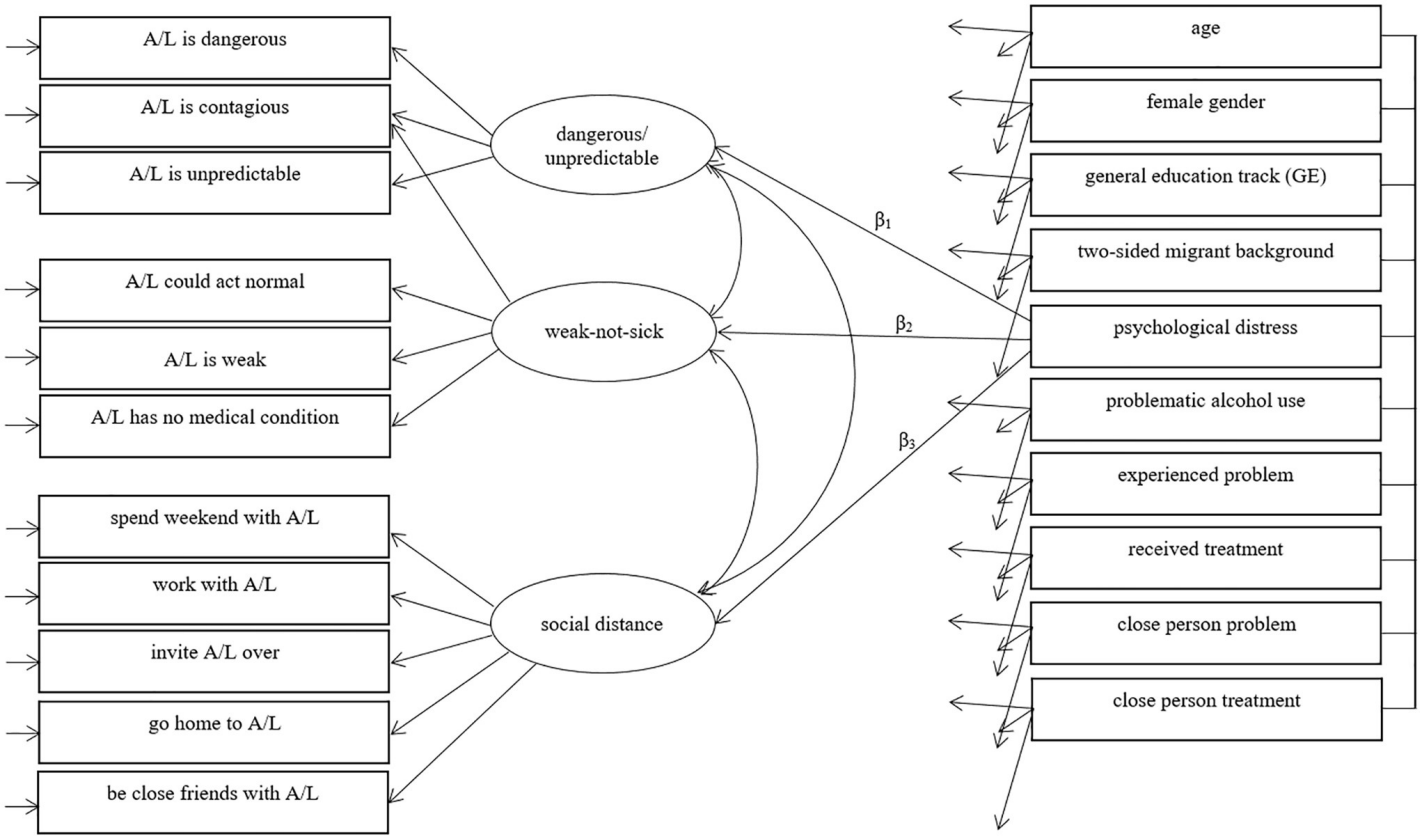
Schematic illustration of the prediction model for all the five vignettes. All predictors (demographic characteristics, current mental health symptoms, exposure variables) predicted the three latent factors. This is illustrated in more detail for the ‘experienced problem’ with the paths β_1_, β_2_ and β_3_. A/L = Anna or Lukas.

Fig 2 shows the prediction model that was fitted and examined for each vignette separately. Model fit increased significantly for all five models after entering the emerging predictors. The fit indices for the five final prediction models (including both initial and emerging predictors) were similar or better than the ones from the original CFA model (Fig 1). The model fit statistics for the five final models were as follows: DEP: χ^*2*^ = 219.22, *df* = 120, *p* < .001; χ^*2*^_*change*_ = 46.21, *df* = 21, *p* = .001; CFI = 0.99; TLI = 0.99; RMSEA = 0.03 (90% CI 0.02–0.04), *p* = 1.000; ALC: χ^*2*^ = 282.77, *df* = 120, *p* < .001; χ^*2*^_*change*_ = 62.21, *df* = 21, *p* < .001; CFI = 0.98; TLI = 0.97; RMSEA = 0.04 (90% CI 0.03–0.04), *p* = 1.000; ALC & DEP: χ^*2*^ = 266.58, *df* = 120, *p* < .001; χ^*2*^_*change*_ = 100.34, *df* = 21, *p* < .001; CFI = 0.97; TLI = 0.96; RMSEA = 0.04 (90% CI 0.03–0.04), *p* = 1.000; SCH: χ^*2*^ = 245.70, *df* = 120, *p* < .001; χ^*2*^_*change*_ = 96.76, *df* = 21, *p* < .001; CFI = 0.98; TLI = 0.97; RMSEA = 0.03 (90% CI 0.03–0.04), *p* = 1.000; and SOC: χ^*2*^ = 255.60, *df* = 120, *p* < .001; χ^*2*^_*change*_ = 46.04, *df* = 21, *p* = .001; CFI = 0.98; TLI = 0.98; RMSEA = 0.04 (90% CI 0.03–0.04), *p* = 1.000.

Table 2 displays standardised path coefficients and standard error for the models with only initial predictors and models with all predictors for each of the five vignettes. Table 3 presents the associations (positive, negative, none) between predictors and latent factors in the final models of the five vignettes, which are discussed subsequently.

**Table 2:** Standardised path coefficients (standard errors) for predictors for stigma factors ‘dangerous/unpredictable’, ‘weak-not-sick’, and ‘social distance’.

		Step 1: initial predictors included			Step 2: initial and emerging predictors included										Total effects
Latent factor	Vignette	Age	Female gender	Two-sided migrant background	Age	Female gender	Two-sided migrant background	General education track	Psychol-ogical distress	Problematic alcohol use	Experienced problem	Received treatment	Close person problem	Close person treatment	R^2^ (R^2^ adjusted)
Dangerous/ unpredic-table	DEP	0.004 (0.048)	**-0.409 (0.094)**	0.151 (0.109)	0.005 (0.047)	**-0.401 (0.094)**	0.145 (0.108)	0.090 (0.100)	**0.089 (0.045)**	**0.200 (0.100)**	**-0.290 (0.117)**	0.028 (0.164)	0.069 (0.118)	-0.115 (0.130)	**0.045 (0.074)**
	ALC	0.027 (0.048)	**-0.453 (0.089)**	**-0.215 (0.107)**	0.028 (0.048)	**-0.449 (0.090)**	**-0.217 (0.107)**	0.128 (0.097)	**0.131 (0.045)**	-0.150 (0.100)	-0.232 (0.156)	0.182 (0.278)	-0.054 (0.109)	0.131 (0.158)	**0.057 (0.058)**
	ALC & DEP	0.037 (0.049)	**-0.554 (0.104)**	0.030 (0.116)	0.036 (0.048)	**-0.539 (0.104)**	0.025 (0.114)	**0.232 (0.103)**	**0.170 (0.052)**	0.013 (0.901)	**-0.301 (0.138)**	0.241 (0.186)	-0.131 (0.132)	**-0.279 (0.142)**	**0.080 (0.119)**
	SCH	0.028 (0.049)	**-0.622 (0.096)**	-0.004 (0.111)	0.028 (0.048)	**-0.610 (0.096)**	-0.002 (0.109)	0.155 (0.102)	**0.132 (0.049)**	0.082 (0.106)	**-0.642 (0.180)**	0.357 (0.233)	-0.173 (0.183)	0.088 (0.192)	**0.098 (0.137)**
	SOC	-0.019 (0.053)	**-0.451 (0.099)**	-0.035 (0.106)	-0.019 (0.052)	**-0.442 (0.098)**	-0.033 (0.104)	-0.078 (0.100)	0.054 (0.049)	**0.239 (0.093)**	**-0.272 (0.119)**	0.000 (0.187)	0.073 (0.128)	-0.028 (0.159)	**0.051 (0.093)**
Weak-not-sick	DEP	-0.084 (0.053)	**-0.506 (0.096)**	**-0.172 (0.087)**	-0.079 (0.049)	**-0.466 (0.090)**	**0.385 (0.105)**	**-0.465 (0.095)**	0.014 (0.045)	0.004 (0.100)	-0.075 (0.115)	-0.240 (0.157)	0.046 (0.125)	**-0.542 (0.124)**	**0.091 (0.241)**
	ALC	-0.077 (0.053)	**-0.557 (0.092)**	**0.230 (0.114)**	-0.074 (0.051)	**-0.532 (0.088)**	**0.220 (0.109)**	**-0.546 (0.093)**	0.037 (0.046)	0.117 (0.098)	-0.071 (0.152)	0.017 (0.315)	-0.100 (0.110)	-0.172 (0.140)	**0.093 (0.176)**
	ALC & DEP	-0.043 (0.049)	**-0.482 (0.095)**	**0.455 (0.107)**	-0.040 (0.045)	**-0.439 (0.088)**	**0.412 (0.098)**	**-0.540 (0.091)**	0.023 (0.048)	**0.204 (0.092)**	**-0.303 (0.112)**	**-0.412 (0.170)**	0.173 (0.111)	**-0.449 (0.120)**	**0.100 (0.263)**
	SCH	**-0.169 (0.052)**	**-0.478 (0.095)**	**0.262 (0.109)**	**-0.157 (0.048)**	**-0.442 (0.089)**	**0.245 (0.101)**	**-0.657 (0.087)**	0.054 (0.043)	0.104 (0.090)	**0.316 (0.146)**	-0.383 (0.202)	0.200 (0.159)	**-0.586 (0.180)**	**0.099 (0.235)**
	SOC	-0.078 (0.053)	**-0.488 (0.105)**	**0.397 (0.103)**	-0.075 (0.050)	**-0.454 (0.100)**	**0.375 (0.097)**	**-0.477 (0.100)**	-0.039 (0.048)	**0.065 (0.096)**	-0.067 (0.110)	-0.362 (0.192)	-0.077 (0.126)	-0.188 (0.170)	**0.095 (0.203)**
Social distance	DEP	0.151 (0.109)	**0.416 (0.114)**	-0.039 (0.097)	-0.021 (0.043)	**-0.170 (0.086)**	-0.039 (0.096)	0.121 (0.089)	-0.013 (0.041)	0.097 (0.090)	-0.149 (0.106)	-0.033 (0.152)	0.035 (0.112)	-0.167 (0.121)	0.008 **(0.030)**
	ALC	0.014 (0.042)	-0.101 (0.084)	**-0.414 (0.096)**	0.014 (0.041)	-0.097 (0.081)	**-0.401 (0.094)**	0.092 (0.088)	0.012 (0.039)	-0.130 (0.085)	**-0.403 (0.131)**	**-0.703 (0.262)**	-0.170 (0.092)	0.112 (0.141)	**0.031 (0.090)**
	ALC & DEP	0.029 (0.042)	**-0.470 (0.088)**	**-0.388 (0.094)**	0.028 (0.040)	**-0.453 (0.086)**	**-0.375 (0.091)**	**0.279 (0.090)**	-0.037 (0.046)	-0.015 (0.091)	**-0.468 (0.118)**	0.186 (0.154)	0.114 (0.108)	**-0.258 (0.115)**	**0.080 (0.144)**
	SCH	0.032 (0.041)	**-0.221 (0.086)**	**-0.201 (0.096)**	0.030 (0.039)	**-0.209 (0.082)**	**-0.191 (0.091)**	**0.277 (0.083)**	**-0.133 (0.040)**	0.157 (0.083)	-0.164 (0.141)	-0.260 (0.217)	-0.285 (0.147)	0.017 (0.162)	0.020 **(0.118)**
	SOC	-0.070 (0.045)	**-0.425 (0.082)**	**-0.246 (0.093)**	-0.069 (0.044)	**-0.416 (0.081)**	**-0.241 (0.092)**	**0.218 (0.084)**	-0.026 (0.041)	0.117 (0.084)	-0.062 (0.103)	0.001 (0.181)	-0.179 (0.106)	-0.046 (0.145)	**0.056 (0.094)**

Significant effects are indicated in bold type. Paths from age and psychological distress are fully standardized coefficients; paths from binary predictors are standardized for the dependent latent variable only. DEP = depression only vignette, ALC = alcohol abuse only vignette, ALC & DEP = Alcohol abuse and depression combined vignette, SCH = Schizophrenia vignette, SOC = Social anxiety vignette

**Table 3:** Associations (positive, negative, none) between predictors and latent factors in the final models (including initial and emerging predictors) for the five vignettes.

		Latent variable		
Predictors	Vignette	Dangerous / unpredictable	Weak-not-sick	Social distance
Age	DEP	n.s.	n.s.	n.s.
	ALC	n.s.	n.s.	n.s.
	ALC & DEP	n.s.	n.s.	n.s.
	SCH	n.s.	-	n.s.
	SOC	n.s.	n.s.	n.s.
Female gender	DEP	-	-	-
	ALC	-	-	n.s.
	ALC & DEP	-	-	-
	SCH	-	-	-
	SOC	-	-	-
Two-sided migrant background	DEP	n.s.	+	n.s.
	ALC	-	+	-
	ALC & DEP	n.s.	+	-
	SCH	n.s.	+	-
	SOC	n.s.	+	-
General education track	DEP	n.s.	-	n.s.
	ALC	n.s.	-	n.s.
	ALC & DEP	+	-	+
	SCH	n.s.	-	+
	SOC	n.s.	-	+
Psychological distress	DEP	+	n.s.	n.s.
	ALC	+	n.s.	n.s.
	ALC & DEP	+	n.s.	n.s.
	SCH	+	n.s.	-
	SOC	n.s.	n.s.	n.s.
Problematic alcohol use	DEP	+	n.s.	n.s.
	ALC	n.s.	n.s.	n.s.
	ALC & DEP	n.s.	+	n.s.
	SCH	n.s.	n.s.	n.s.
	SOC	+	+	n.s.
Experienced problem	DEP	-	n.s.	n.s.
	ALC	n.s.	n.s.	-
	ALC & DEP	-	-	-
	SCH	-	+	n.s.
	SOC	-	n.s.	n.s.
Received treatment	DEP	n.s.	n.s.	n.s.
	ALC	n.s.	n.s.	-
	ALC & DEP	n.s.	-	n.s.
	SCH	n.s.	n.s.	n.s.
	SOC	n.s.	n.s.	n.s.
Close person problem	DEP	n.s.	n.s.	n.s.
	ALC	n.s.	n.s.	n.s.
	ALC & DEP	n.s.	n.s.	n.s.
	SCH	n.s.	n.s.	n.s.
	SOC	n.s.	n.s.	n.s.
Close person treatment	DEP	n.s.	-	n.s.
	ALC	n.s.	n.s.	n.s.
	ALC & DEP	-	-	-
	SCH	n.s.	-	n.s.
	SOC	n.s.	n.s.	n.s.

N.s. = not significant path coefficient, + = positive path coefficient,— = negative path coefficient.

DEP = depression only vignette, ALC = alcohol only vignette, ALC & DEP = Alcohol and depression combined vignette, SCH = Schizophrenia vignette, SOC = Social phobia vignette

#### Initial predictors

Age was not significantly associated with stigma in most vignettes, except for the SCH vignette, where older students characterized Anna/Lukas as sick rather than weak more than younger students. Being female was associated with having less stigmatizing attitudes. Female students evaluated the character as being less dangerous/unpredictable and more sick rather than weak and also indicated that they would not socially distance themselves from the character to such an extent as male students, except for the ALC vignette (where there was no significant gender difference on the latent variable ‘social distance’).

Opposing associations were found between two-sided migrant background and the latent factors. Students with a two-sided migrant background were more likely to perceive the character as weak in all vignettes, but less likely to socially distance themselves in all but the DEP vignette compared to students with Swiss origins. Furthermore, students with a two-sided migrant background were less likely to characterize the character as dangerous/unpredictable than students of Swiss origin for the ALC vignette.

#### Emerging predictors

Education track was also associated with stigma, although the directions of these associations differed for the three latent factors. Students following the GE-track were less likely to characterize the character as weak rather than sick (all vignettes), but more likely as dangerous/unpredictable (ALC & DEP-vignette) than VET-students. Students following the GE-track were also more likely to socially distance themselves from the character of the ALC & DEP, the SCH and the SOC vignette.

Students with higher levels of psychological distress perceived the characters of all but the SOC vignette as more dangerous/unpredictable compared to students who reported less psychological distress. However, for the SCH vignette, higher levels of psychological distress were associated with less social distance.

Students who reported problematic levels of alcohol use were more likely to stigmatize the character in three of five vignettes than students who reported a non-problematic alcohol use. Compared to non-problematic alcohol users, problematic alcohol users perceived the character of the DEP and SOC vignettes as more dangerous/unpredictable. Furthermore, they characterized the character of the ALC & DEP vignette as being weak.

Generally, having experienced a similar problem to the character was predictive of less stigmatizing attitudes to four of five vignettes. Students who had experienced a similar problem compared to those who did not have such experience or did not want to tell, were less likely to perceive the character of all vignettes except the ALC vignette, as being dangerous/unpredictable. For the ALC and ALC & DEP vignettes, this same group of students reported that they would also socially distance themselves to a lesser extent from the character. Students who had experienced a similar problem to that described in the ALC & DEP vignette characterized the character as less weak than those who had never experienced something similar. In contrast, students who had experienced a similar problem to that described in the SCH vignette were more likely to describe it as a personal weakness rather than sickness. In contrast to having experienced a similar problem oneself, knowing someone within the close circle who had suffered from one of the five conditions was not significantly associated with stigma.

Students who received professional help for their alcohol-associated problems and students who knew someone in the close circle who had received professional help for co-existing alcohol abuse and depression problems were less likely to socially distance themselves compared to those who were not exposed to professional help. Furthermore, students who personally received treatment or knew someone close who had treatment for co-existing alcohol abuse and depression problems, characterized the character as being sick rather than weak relative to those with no such exposure. This was also the case for students who knew someone who had received professional help for depression or schizophrenia; they also perceived the problem less as a weakness than as an illness. Lastly, these students were less likely to perceive the character in the ALC & DEP vignette as dangerous/unpredictable.

#### Summary of the results

The associations between predictors and stigma in the final models can be summarized as follows: Female gender was associated with less stigmatizing attitudes, whereas age was generally not identified as significant predictor. In terms of educational track and migration background, opposing associations were identified with different latent variables. Current mental health symptoms were mostly–if at all–associated with more stigmatizing attitudes, whereas negative relationships were found between some of the exposure variables and the latent variables. It needs to be pointed out that the variance explained by the models was rather small: the adjusted R^2^ varied between 0.03 and 0.26.

## Discussion

The current study investigated the factor structure and predictors of stigmatizing attitudes in a representative sample of adolescents and young adults from the German-speaking part of Switzerland. CFA indicated that a three-factor model–consisting of the latent variables ‘dangerous/unpredictable’, ‘weak-no-sick’, and ‘social distance’–best fitted the data, corresponding to earlier research [27–29]. Predictors for the identified latent factors were examined separately for each of the five vignettes. Adding a broad range of background, experience and exposure variables to the prediction model improved model fit compared to models that included only fundamental, initial predictors (e.g., age, gender).

We consider firstly, the results concerning the initial predictors (gender, age, migration status), followed by a discussion of emerging predictors (academic track, current mental health status and exposure). No significant associations were found with age. This is likely to reflect the relatively narrow age range of the study. However, clear and substantial effects of gender were identified: the current study indicated that females stigmatize peers with mental disorders to a lesser extent than males. This pattern was–with one exception–identified for all latent variables and across all vignettes. Similar gender differences were reviewed in an article by Kaushik et al. [8] that specifically focused on youth. These authors reasoned that these differences might be an effect of the widely held belief that males should be able to handle their mental health problems on their own. While such an explanation might be valid for the ‘weak-not-sick’ factor in the current study, it seems less plausible for the other latent variables. This is particularly the case for the ‘dangerousness/unpredictable’ factor, since the finding that males perceived the character in the vignette as more dangerous/unpredictable compared to females contradicts the stereotypical image of masculinity. However, taking into account that males with mental disorders are typically more stigmatized than females [8, 10, 36], it is possible that the consistently-found higher stigmatizing attitudes among male participants was also a consequence of the methodological design of the current survey, given that the character’s gender in the vignette was matched to the gender of the participant (i.e., male participants read vignettes that portrayed a male, whereas the character that was presented to female participants was a female).

In terms of educational stream (an emerging predictor) and migration background (initial predictor), opposing associations were identified with different latent variables. This divergence was most pronounced for the factors ‘weak-not-sick’ and ‘social distance’, even though these latent variables were not negatively correlated. While participants with a higher educational level (i.e. those following GE) as well as those with a Swiss origin were less likely to perceive the character in the vignette as weak rather than sick, they were more likely to socially distance themselves from the person. Different (cultural) beliefs of parents that might have been adopted by the adolescents and young adults who participated in our study might have led to these findings. Particular subgroups with a migration background or with a lower socioeconomic status might, for instance, conceptualize the lack of resilience and weak character as a cause of mental illness, which does not necessarily translate into their desire to socially distance themselves from an affected individual. However, due to the heterogeneity of the studied subgroups (e.g., no differentiation of the type of school that was followed in the GE- or VET-track and no differentiation of the various migration backgrounds), these assumptions remain speculative at this point.

In terms of exposure variables, the current study found that some of these predictors were associated with lower levels of stigmatizing attitudes in some latent variables and in regard to particular vignettes, which is consistent with some review articles [9, 10, 12] and individual studies with a similar methodology (e.g., [28, 37]). Only the exposure variable, ‘knowing someone close who had a similar problem as the character in the vignette’, was not associated with any of the latent variables in the current study. This might have been due to the fact that the survey did not ask about the nature and quality of the contact with the person with a similar problem. Hence, it is possible that negative and positive experiences of participants who stated that they knew someone close with a mental disorder might have cancelled each other out, leading to the reported null finding. In contrast, knowing someone close who has received treatment might have been positively associated with some of the latent variables, since portrayals of successful treatment of people with mental disorders seem to reduce stigmatizing attitudes [38]. Hence, personally knowing someone with a mental disorder or someone who has received treatment for such a condition might only have a destigmatizing effect if this personal contact challenges rather than reinforces the stereotypes of a person [39]. Subsequent studies should therefore assess the nature of such exposure in greater detail.

In contrast to the measures of exposure used here, where the participant had to explicitly confirm that they ever had a problem similar to that described in the vignette, the self-reported assessment of current mental health symptoms by means of the K6 (assessing psychological distress, similar to the symptomatology described in the DEP vignette) and AUDIT-C (assessing problematic alcohol use, similar to the symptomatology described in the ALC vignette) can also be understood as the participant’s implicit assessment of their current and immediate ‘exposure’ to their own mental health problems. In the current study, these implicit exposure measures were mostly–if at all–associated with more stigmatizing attitudes. More precisely, higher psychological distress increased the perception that the character in the vignette was dangerous and unpredictable across all vignettes, except for SOC. Furthermore, problematic alcohol use was related to greater stigmatizing attitudes in some vignettes in the latent variables ‘dangerous/unpredictable’ and ‘weak-not-sick’. In contrast, and as mentioned above, explicitly assessed exposure was–if at all–mostly associated with less stigmatizing attitudes. This discrepancy between explicit and implicit exposure variables might arise because an explicit confirmation of a mental health problem requires a certain degree of insight and literacy on the part of the participant (unless someone else such as a health professional identified a particular mental disorder), whereas this is not a necessity when rating individual symptoms of psychological distress or alcohol use. An explicit confirmation of a mental health problem might go hand in hand with greater empathy and less stigmatizing attitudes towards people who are suffering from mental disorders. In contrast, youth with heightened levels of psychological distress or problematic alcohol use might not perceive themselves as similar to the group of peers with particular mental disorders, but rather stigmatize them in order to clearly differentiate themselves from the group of ‘others’. Furthermore, it needs to be considered that the explicit exposure might have occurred in the distant past, whereas the implicit exposure questions referred to a more current time frame (the K6 referred to the past 30 days, the AUDIT-C to the past 12 months). Currently experiencing symptoms of mental health problems might have a negative impact on personal stigmatizing attitudes through self-stigmatization, i.e. through turning prejudice against oneself [1]. However, if this were the case, more vignette-specific associations would have been expected (heightened psychological distress: more stigmatizing attitudes in the DEP- and ALC & DEP-vignettes; problematic alcohol use: more stigmatizing attitudes in the ALC- and ALC & DEP-vignettes), which was not confirmed by the data. Another possible explanation for the association between heightened psychological distress and more stigmatizing attitudes can be based on perception biases [40, 41]. Youth with heightened psychological distress might generally have a more negative perception of their world, which eventually contributed to the higher stigmatizing attitudes of this group in the latent variable ‘dangerousness/unpredictability’.

There are only a few other studies that looked at associations between psychological distress or depressive symptoms and stigmatizing attitudes [14, 42–44]. The findings of these studies are themselves conflicting, possibly reflecting differences in measures used to assess such mental health symptoms. However, a study from Australia that surveyed adults and used the K10 (a longer form of the K6) found that psychological distress was associated with increased personal depression stigma, which is consistent with our results in a younger sample. Depressive symptoms (as measured by the CES-D) were not related to personal depression stigma in the study mentioned, leading the authors to speculate that the K10 might tap aspects (of psychopathology) beyond depressive symptoms that might be of particular importance to personal stigma. Similarly, the specific questions of the K6 might also have contributed to the associations between heightened levels of psychological distress and more stigmatizing attitudes in our study.

In terms of problematic alcohol use, we could identify only one study that included such a predictor [43]. This study did not find significant associations between the AUDIT-C and personal stigma regarding seeking mental health treatment in college students. That these results deviated from our findings may have been due to assessing stigma towards help-seeking rather than towards individuals with particular mental disorders themselves. Hence, there is a need for further studies that examine the associations between problematic alcohol use and stigma towards mentally-ill individuals.

The following limitations must be considered when interpreting the results of the present study. Most importantly, stigmatizing attitudes were assessed in the context of hypothetical scenarios (i.e., no attitudes or actual behaviour towards real-life people were measured). Furthermore, the analyses were based on self-reports, which may have been biased in some instances. However, biases such as social desirability were kept to a minimum by conducting an anonymous online survey. In terms of the analyses, it must be considered that they were based on cross-sectional data. Thus, no casual conclusions can be drawn. Due to the inclusion of numerous predictors in the current study, some multiple category predictors had to be collapsed (e.g., for migration background) in order to achieve model convergence. It must be acknowledged that the groupings of participants (e.g., those with a two-sided migrant vs. Swiss background; those who were following the VET- vs. GE-track) may not be homogenous (as mentioned above). A more fine-grained assessment of some predictors should also be considered in subsequent studies. It should, for instance, not only be established whether a participant had personal experiences with someone close who suffered from a mental disorder. Rather, the quality and the point in time of this experience should be assessed as well. In order to study gender differences, subsequent studies should also consider randomly presenting female and male characters to participants, since the current methodology limited conclusions about the associations between gender and stigmatizing attitudes (see above). Furthermore and as mentioned above, it must be emphasized that the variance explained by the models was rather small. This, and the magnitude of correlations of stigma measures and predictors is comparable to the magnitude of effects found in other settings (e.g., [9, 14, 28, 36, 37, 44]). While the findings may be of some assistance in focusing targets for intervention, our work suggests the need to cast a wider net in searching for determinants of stigma. Lastly, it must be acknowledged that only schools from the German-speaking parts of Switzerland were surveyed. Hence, results may not be generalizable to the other language regions in Switzerland or beyond.

## Conclusions

In the present study, contact with someone close who has received treatment for their mental health problem was associated with lower levels of some stigmatizing attitudes. Being aware that mental disorders (including substance-related conditions) can be treated successfully may have contributed to such a destigmatisation [38]. Hence, enabling such contacts might be a fruitful component of interventions that aim at tackling the stigma associated with mental disorders. The current study further highlights that tailored interventional programs for specific subgroups may be needed. It can, for instance, be assumed that the stigma process differs for youth with different social or ethnic backgrounds, or for students following different academic tracks. However, in order to develop such tailored destigmatisation programs it will first be necessary to gain a deeper understanding of the factors leading to these group differences. Besides these practical implications, the current study also allows us to draw scientifically relevant conclusions. Most importantly, the results on the one hand underpin the importance of conceptualizing public-personal stigma as a multidimensional construct, especially due to the identified opposing associations between some predictors and the different dimensions of stigma identified in our confirmatory factor analyses. On the other hand, the findings highlight diverging associations between explicit and implicit measures of exposure and stigmatizing attitudes. Studying such differences more systematically may further deepen understanding of hitherto contradictory results.

## Supporting information

S1 File (INP)

S2 File (INP)

S3 File (INP)

S4 File (INP)

S5 File (INP)

S6 File (INP)

S7 File (INP)

S8 File (INP)

S9 File (INP)

S10 File (INP)
